# Predictive Factors for Oral Immune Modulation in Cow Milk Allergy

**DOI:** 10.3390/nu14030494

**Published:** 2022-01-23

**Authors:** Ioana Adriana Muntean, Ioana Corina Bocsan, Lena Katharina Wiest, Irena Pintea, Carmen Teodora Dobrican, Emanuela Duca, Corina Ureche, Anca Dana Buzoianu, Diana Deleanu

**Affiliations:** 1 Department of Allergology and Immunology, “Iuliu Hatieganu” University of Medicine and Pharmacy, 400012 Cluj Napoca, Romania; Adriana.Muntean@umfcluj.ro (I.A.M.); irena.nedelea@umfcluj.ro (I.P.); dobrican.carmen@umfcluj.ro (C.T.D.); diana.deleanu@umfcluj.ro (D.D.); 2 Department of Allergology, “Octavian Fodor” Institute of Gastroenterology and Hepatology, 400162 Cluj Napoca, Romania; 3 Department of Pharmacology, Toxicology and Clinical Pharmacology, “Iuliu Hatieganu” University of Medicine and Pharmacy, 400337 Cluj Napoca, Romania; abuzoianu@umfcluj.ro; 4 Department of Anesthesiology, Intensive Care, Emergency and Pain Medicine, Hospital of Villingen-Schwenningen, 78056 Villingen-Schwenningen, Germany; lenaboehme@web.de; 5 Department of Pediatric Pneumology, Pediatric Hospital, 400371 Cluj Napoca, Romania; emma_floca@yahoo.com; 6 First Internal Medical Department, “George Emil Palade” University of Medicine, Pharmacy, Sciences and Technology, 540139 Târgu Mureș, Romania; corina.ureche@umfst.ro

**Keywords:** casein, cow milk allergy, oral immunotherapy, oral tolerance

## Abstract

Aim: The present study analyzed clinical and biological factors that might predict achievement of tolerance in patients with IgE-mediated cow milk allergy (CMA). Method: Seventy patients with IgE-mediated CMA (44.24 ± 24.16 months) were included in the study. The patients were evaluated clinically through skin prick test and sIgE to whole milk, casein, beta-lactoglobulin and alpha-lactalbumin. An eviction diet of 6 months was established, followed by oral food challenge test (OFC) and oral immunotherapy (OIT) with baked milk for 6 months. The tolerance was assessed after 2 years follow up. Results: Thirty percent of patients presented anaphylaxis of different degrees of severity as first manifestation of CMA. Sixty-two patients followed OIT or an accelerated reintroduction of milk. Ten patients (14.28%) did not obtain tolerance to milk within 2 years. A larger wheal in SPT and higher sIgE to milk, casein and betalactoglobulin were noted in patients with positive OFC. A basal level of <2.5 kU/l for sIgE to milk and <11.73 kU/l for sIgE to caseins predicted the occurrence of tolerance in patients with all types of clinical manifestations, including anaphylaxis. Conclusion: Basal levels of sIgE to milk and casein may help to identify patients that could become tolerant to milk.

## 1. Introduction

Cow milk allergy (CMA) is the most common food allergy in children, with an estimated prevalence of 0.5% to 3% in the pediatric population below 1 year old [1]. Self-reported incidence of CMA is much higher than confirmed allergy in both children and adults [2]. The incidence of self-reported allergy varies between 1.2% to 17%, while the rate of prevalence for milk allergy confirmed by an oral food challenge test is lower, between 0% and 3% [3]. The sensitization to milk is less than 1% in the general population, varying throughout Europe, [4,5]. When the confirmation of the allergy is obtained through skin prick test and specific IgE, the prevalence of CMA is between 2–9% [4,5,6]. 

The term cow milk allergy refers to an immune-mediated reaction induced by exposure to cow milk and includes three categories of diseases: IgE-mediated, non-IgE-mediated and combined (produced by IgE and non-IgE mechanisms). An IgE-mediated cow milk allergy is a type I hypersensitivity reaction, and the clinical manifestations occur within minutes to 2 h after milk ingestions. This form represents almost 60% of CMA cases, but this estimation could vary according to patient age and geographical area [1,7].

The clinical manifestations are variable from acute urticaria or exacerbation of atopic dermatitis to the most severe presentation, which is anaphylaxis [1]. CMA is responsible for 10–19% of all food-induced anaphylactic cases, being the third cause of anaphylactic reactions induced by foods, after peanuts and tree nuts [8]. A positive diagnosis algorithm starts with a careful clinical evaluation, followed by skin prick test and laboratory findings [8]. Skin prick test with fresh milk or standardized extract represents a fast method to detect sensitization but not the allergy [9]. Measurement of specific IgE to cow milk through ELISA, RAST or CAP-FEIA technique is a diagnostic approach with high sensitivity, but sometimes, it may deliver false positive results; thus, they should be analyzed in the clinical history context. A basophil activation test is another useful diagnostic tool for CMA in combination with sIgE, especially in children with atopic dermatitis [10], but it is not frequently used in clinical practice. There are still no equal test assay systems for serum sIgE, which makes for a difficult comparison between studies and techniques. Several studies have tried to describe the predictive values of IgE levels for clinical reactivity [8], but the differences are quite significant mainly due to various selection criteria, age of the patient or different criteria for analyzing the clinical reactivity [9,11,12]. 

The first measure in the management of CMA is allergen avoidance. A diet without milk and dairy products is recommended until clinical tolerance is induced [2,6,13]. The oral tolerance to cow milk is reached in almost half of the patients by an age of 5 years, increasing the rate up to 75% until teenage years [6,14], but some patients remain with persistent CMA [13,15]. However, the experience of the last 20 years has shown that the natural history of food allergy is changing and that less individuals become tolerant and that a longer time to resolution is needed [13].

Oral immunotherapy (OIT) has shown some promising results in improving patients’ quality of life in CMA. It is a therapeutic method that can also be used in young children. Adverse reactions including anaphylaxis may occur during OIT, especially during the escalation phase. The rate of desensitization is variable, with 20–30% of patients remaining with persistent CMA despite OIT [6]. The tolerance to cow milk induced by OIT or achieved naturally may vary from country to country, and it is influenced by the genetic inheritance and the microbiota from the gut [16]. OIT may permit achievement of a rapid tolerance to milk, which allows the children to have normal activities without any restrictions. Standardized protocols of OIT with validated optimal dose and ideal duration, data regarding degree of protection, safety, and efficacy in different ages and populations need to be established [17,18,19]. There is also an urgent need to establish standardized outcome measures to be applied in food allergy studies, for both prediction of tolerance and for monitoring of OIT [20]. This may allow for a better harmonization of data resulting from different clinical trials.

The aim of the present study was to identify possible clinical and biological predictive factors for achievement of tolerance after OIT in a cohort of patients with IgE-mediated CMA. The second objective was to establish the effectiveness of a modified protocol for oral immunotherapy to milk in obtaining oral tolerance. 

## 2. Materials and Methods

### 2.1. Patients and Study Design

The study was an analytic, transversal study. The present research analyzed clinical and biological factors that might predict the occurrence of tolerance in patients with cow milk protein allergy. 

Seventy-six patients with milk-induced reactions presented for allergological evaluation. The patients were evaluated at the Allergology Department of Regional Institute of Gastroenterology and Hepatology “Prof. Dr. Octavian Fodor” in Cluj Napoca and at the Almedo Clinic in Cluj Napoca between January 2013–November 2021. Only patients with an unequivocal positive immediate allergic reaction after contact with cow milk as well as documented evidence of sIgE to cow milk protein by blood tests and/or a skin prick test were included. The exclusion criteria were: non-IgE-mediated hypersensitivity reactions induced by cow milk, patients without a definitive positive diagnosis of CMA, patients that refused to sign the informed consent, and patients for which follow up was not performed. Based on these criteria, six patients were excluded from the final analysis. 

Seventy patients with IgE-mediated CMA that had presented for allergological evaluation were included in the study. The mean age was 44.24 ± 24.16 months when the patients were included in the evaluation, and the sex ratio was M:F = 1.41. Diagnosis of IgE-mediated CMA was established according to international guidelines, based on history, clinical evaluation, skin prick test (SPT) and sIgE to milk and components. 

The study protocol was approved by the University Ethics Committee of the University of Medicine and Pharmacy (293/28 July 2013), according to the principles from Declaration of Helsinki. Each patient signed the informed consent before the study began. 

### 2.2. Allergological Evaluation

**Figure 1:**
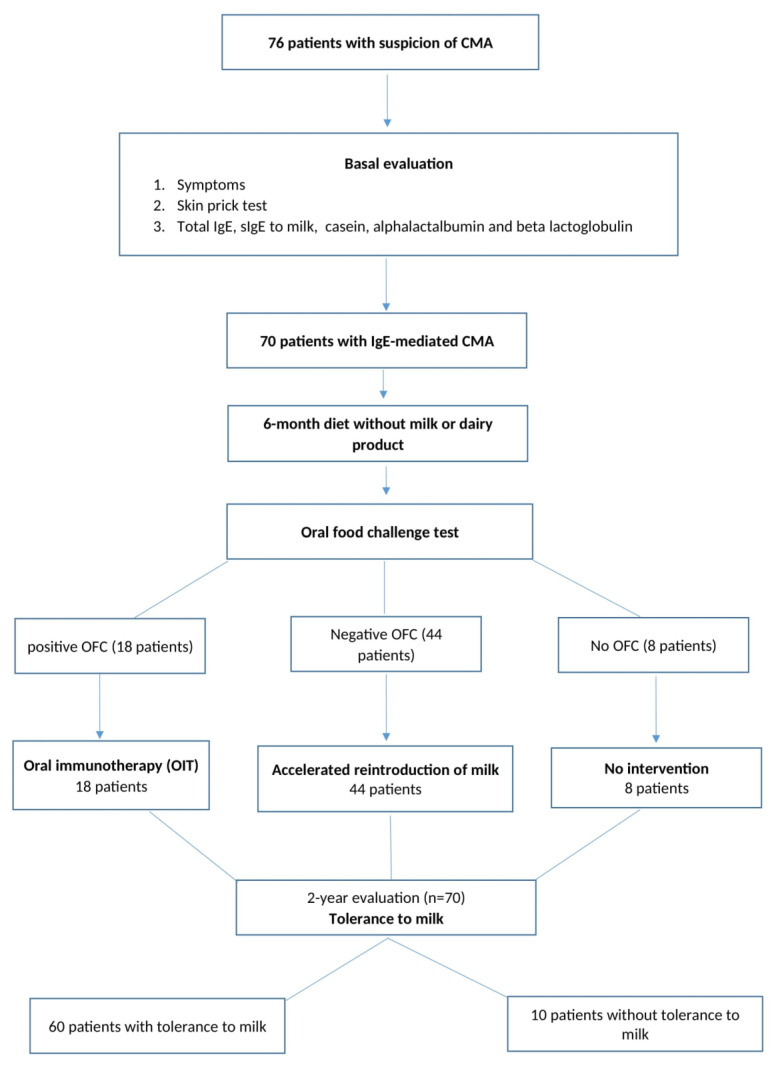
The algorithm of evaluations and therapeutic interventions in patients with cow milk allergy.Abbreviations: CMA, cow’s milk allergy; IgE, immunoglobulin E; OFC, oral food challenge.

Clinical evaluation was performed at the beginning, when the patients were included in the study (see Figure 1). From anamnesis, the following demographic and clinical data were recorded: age, gender, living area (urban/rural) and clinical picture of the first allergic reaction, onset of disease, duration until first allergological diagnosis, family history of atopy, and other allergic diseases associated.

Skin prick of milk protein mix was performed. Skin prick tests were positive if the wheal diameter was ≥3 mm compared to the negative control. Standardized allergen extracts (Hal Allergy, Netherlands) were used. The value in mm was recorded as a medium diameter wheal size.

Serological tests implied determination of total Ig, specific IgE for cow milk (whole extract) and casein, beta-lactoglobulin and alpha-lactalbumin. Laboratory test results were obtained through electrochemiluminescence immunoassay method (ECLIA).

The atopy diagnosis was established through skin prick test at enrollment, according to international guidelines [21]. The skin prick test included the following panel of allergens: house dust mites (Derm. Pteronyssinus and Derm. Farinae), pollens (grasses, cereals, birch and weeds), animal dander (cat and dog) and molds (Alternaria alternata). 

After the positive diagnosis was established, a diet without milk or dairy products was recommended for 6 months. After 6 months, the remission of symptoms or an accidental exposure to milk were assessed. An oral challenge test with milk 3.5%, baked for 30 min was performed in 62 of patients. The positivity of OFC was established if the patient had a clinical manifestation and if the quantity of milk that induced the reactivity was noted. Simple-blind OFC was not performed in patients if the parents refused to sign the informed consent. The simple blind OFC protocol included 4 steps:

1. 2 mL rice milk (commercially available) as placebo;
2. 0.25 mL baked cow milk plus 1.75 mL of rice milk;
3. 0.5 mL baked cow milk plus 1.5 mL of rice milk;
4. 1 mL baked cow milk plus 1 mL of rice milk.

All the doses were given at 30 min time intervals. The OFC was considered positive if the patient presented clinical manifestations in the aforementioned 4 steps. The OFC was considered negative if the patients tolerated 1 mL milk. If the patients tolerated 1 mL milk during OFC, they continued with an accelerated reintroduction of baked milk (see Figure 1 and Table 1) to reach, in 48 h, the maintenance dose of milk that was used in the protocol of oral immunotherapy. The rapid reintroduction of baked milk was performed in the allergological department under medical supervision until the maintenance dose of 200 mL was reached.

### 2.3. Oral Immunotherapies

A group of patients (18 patients) underwent open oral immunotherapy (see Figure 1). The procedure consisted of the administration of progressively increasing amounts of baked milk 3.5% to induce tolerance and to reduce the allergic symptoms until disappearance. Small amounts of baked milk were administered sublingually initially, with an increasing amount administered orally according to tolerance (build up phase period), to a dose that was given daily (maintenance period) continuously. The initiation of immunotherapy was performed in a specialized allergology unit with existing facilities for emergency assistance if the patients developed adverse reactions. The protocol started with 0.05 mL baked milk, and the maintenance dose of 50 mL was supposed to be reached in 3 weeks. In some patients, the induction phase lasted more than 6 months until the maintenance dose was reached. When the patients were in the maintenance phase, they were allowed to introduce milk substitutes such as yoghurt, cream or ice cream. The protocol of updosing is presented in Table 1. The acquisition of tolerance was established after 2 years follow up.

### 2.4. Statistical Analysis

Statistical analysis was carried out using the MedCalc Statistical Software version 18.10 (MedCalc Software bvba, Ostend, Belgium; http://www.medcalc.org; (accessed on 20 November 2021). Quantitative data were evaluated for normality of distribution using the Kolmogorov–Smirnov test. They were characterized by median and 25–75 percentiles. Qualitative data were expressed as frequency and percentages. Comparisons between groups were performed using the Mann–Whitney or chi-square tests whenever appropriate. The correlation between variables was established using Spearman’s correlation. ROC curves were used in order to find cut-off values for quantitative variables that could discriminate between patients with a tolerance to milk and those without. A *p* value of <0.05 was considered statistically significant.

## 3. Results

Seventy patients with IgE-mediated CMA were evaluated (Table 2). Most of the patients (62 patients, 88.6%) followed a rapid reintroduction of milk or OIT for milk. Ten patients (14.28%) did not obtain tolerance to milk within 2 years after the first evaluation and positive diagnosis of cow milk allergy. Only two patients (11.1%) from the group that followed OIT did not gain oral tolerance in this interval of time.

Demographic data are presented in Table 2.

CMA was noted more frequently in boys than in girls, and more females obtained tolerance after OIT than males (93.1% vs. 80.5%), but the difference was not statistically significant. Twenty-nine patients (41.4%) had a positive family history of atopy, but this did not influence the induction of tolerance compared to patients without a family history of allergy. Personal history of respiratory and/or food allergy were noted in almost one-third of the patients, without any influence in obtaining tolerance. Twelve patients (60%) with other food allergies tested positive to egg, followed by peanuts and other nuts. 

The average duration of disease from the onset of the symptoms until the positive diagnosis of CMA was 20 (6.5–40.75) months, and a longer time was noted in patients with persistent allergies compared to those with a tolerance to milk (43.5 (21.5–112.5) vs. 18 (4.5–36), *p* = 0.027). The family or personal history of allergy or the severity of first clinical presentation did not accelerate the presentation to a specialist for evaluation of CMA.

### 3.1. Clinical Manifestations

The analysis of clinical manifestations revealed that the symptoms occurred, on average, at the age of 9 months (9.72 ± 4.66 years, minimum 1 month, maximum 24 months). The age of onset was higher in patients with persistence of CMA. 

Thirty percent of the patients (21 pts) presented anaphylaxis of different degrees of severity. Most of the patients (65.7%) presented cutaneous manifestation such as acute urticaria or aggravation of atopic dermatitis or both (Table 3). The clinical manifestation at the onset of the allergy did not predict the occurrence of tolerance to milk.

### 3.2. Skin Prick Test and sIgE

Skin prick test and specific IgE to milk and major proteins were performed in all cases. The size of the wheal was higher in patients with persistent allergy, but the difference did not reach the level of statistical significance. Basal median values of specific IgE to milk and to casein were significantly higher in patients without oral tolerance (Table 4).

The basal results of skin prick test and laboratory values were also analyzed in relation to clinical reactivity after OFC. The oral food challenge test was performed after a period of 6 months of eviction diet in order to establish the opportunity of oral immunotherapy. OFC was performed in 62 patients (88.57%), and it was positive for 18 of them (Table 5). The clinical reactivity during OFC was more frequently noted in patients with persistent CMA (*p* = 0.003).

The patients with positive OFC had significantly higher values of specific IgE to milk (*p* = 0.017), casein (*p* = 0.006), and beta lactoglobulin (*p* = 0.011), but not to alpha-lactalbumin (*p* = 0.083) compared to patients with negative OFC. The size of the wheal at skin prick test was also significantly higher in those patients (*p* = 0.002).

### 3.3. Analysis of Patients with Anaphylaxis Induced by Cow Milk Proteins

Twenty-one patients with CMA presented anaphylaxis grade 2 to 4 of severity, from which three patients (14.28%) had a persistent allergy to cow milk. The anaphylaxis as a primary manifestation of CMA was not correlated with a personal history of allergy to other foods or respiratory allergens (*p* = 1, respectively *p* = 0.74) and to a familial history of atopy (*p* = 1). Oral immunotherapy was performed in 18 patients, and all of them obtained tolerance compared to those patients that had a persistent form of CMA and did not follow OIT (*p* = 0.001). The severity of initial anaphylactic reactions did not predict de-occurrence of oral tolerance (*p* = 0.792) after OIT.

Specific IgE to milk and casein wase significantly higher in patients with anaphylaxis and persistent allergy to cow milk compared to those who obtained oral tolerance (Table 6).

The ROC curves for basal values of specific IgE for milk and casein were analyzed, and the cut-off values were calculated for these parameters in relation to the presence of tolerance after 2-year follow up after the onset of OIT. The cut off values, AUC, and sensitivity and specificity are presented in Table 7. 

During OIT, no severe reactions were noted. Few patients presented mild skin eruptions or perioral contact dermatitis with spontaneous remission or after administration of H1 antihistamines. None of the patients presented bronchospasm, diarrhea or anaphylactic reactions that needed administration of epinephrine.

## 4. Discussion

The present study assessed the clinical and biological changes in patients with IgE-mediated CMA, showing that both sIgE to milk and casein basal levels could predict the occurrence of oral tolerance after OIT or after rapid reintroduction of milk. The study also demonstrated the efficacy of a modified protocol for oral immunotherapy in inducing oral tolerance to milk.

Cow milk allergy is a common allergy in the pediatric population, being the first food allergy described in the allergic march [2,22]. It may be over- or underdiagnosed, depending on the type of evaluation. Some health care professionals, but especially parents, confuse CMA with lactose intolerance, leading to inappropriate diets. Even if true CMA is diagnosed, the type of elimination diet, substitutive products and the duration of such elimination are not always logical. Complete elimination of cow milk without an appropriate substitution can lead to growth impairment, malnutrition, and deficiencies in nutrients with long term consequences [22]. Food allergies negatively affect quality of life for children and their parents, with a significant disruption in family life and social interactions [23,24,25]. Both physicians and parents should understand the multifaceted clinical and biological aspects of CMA to know how to manage further diets.

In the present study, the onset of CMA was noted in the first year of life in few patients, with the first symptoms being described afterward, but no later than the age of 2 years. CMA is mostly a disease of infancy and early childhood. Most of the studies reported that affected children presented symptoms within the first 6 months of life and sometimes earlier, usually before 1 month of age and often within 1 week after the introduction of cow milk proteins to their diet [15,22,26]. In the present study, the average onset of CMA was 9 months, later than in the previous studies [27,28], but all of the patients had clinical manifestations within 2 years of life. Boys were more affected by CMA than girls, similar to the EuroPrevall study [29]. The family history of atopy was reported in more than 40% of the patients, as in the EuroPrevall study [29], but the percentage reflects global atopy in mothers and fathers and is not separated by gender. 

Eight patients from the countryside are not enough to make proper conclusions about a difference in CMA between patients living in the cities and those living in the countryside. For future studies, an overall online database should be created for doctors from different departments in order to introduce patients with CMA, especially when small sample sizes are present. Nevertheless, a long period from the first symptoms until the first allergological consultation occurs (median 20 months) is unacceptable. It shows that neither doctors nor the parents are aware of food-induced allergies or comorbidities commonly associated with CMA (e.g., acute urticaria, atopic dermatitis, anaphylaxis, and GERD), and further efforts are needed in order to improve the situation in Romania. Mainly, pediatricians and family doctors must be aware of this topic and should refer probable cases to an allergology department for further evaluation. It is especially important for severe cases presenting clinically with anaphylactic reaction to have proper management and to prevent further acute episodes. Patients with anaphylaxis had earlier presentation to an allergologist (median 10 months), showing that a severe reaction may increase the anxiety of both children and parents and may make them aware of a potential risk. The specialist visit should occur as soon as possible in order to reduce the sequelae, an improper diet, and in order to provide a proper treatment regimen as well as possible oral immunotherapy for patients.

The majority of children with CMA had one or more symptoms that involved one or more organs, mainly the gastrointestinal tract and/or skin. More than half of the patients had skin manifestations (acute urticaria, aggravation of atopic dermatitis, contact dermatitis) as the first manifestation of CMA. Digestive symptoms alone were described only in three patients (4.2%), which is less frequent than in other studies, but digestive symptoms are more common in non-IgE-mediated reactions to milk [29]. Anaphylaxis as the first manifestation was present more frequently in the present analysis (30% of children) compared to previous data [1,8]. An anaphylactic reaction might increase anxiety in the family, allowing parents to be more aware of the risk of a severe reaction. Patients with milder skin reactions probably skip evaluation in the allergology department and are thus treated by a generalist, pediatrician or dermatologist, which may also explain the lower rate of cutaneous manifestations described in this cohort compared to previous data [8,22,29]. 

Following the ESPGHAN algorithm [30] for the evaluation of children with suspicion of CMA, a simple-blind OFC test was performed in 88.57% of the patients to establish if the patients obtained a tolerance to milk and to assess the opportunity of OIT. OFC should be a part of the routine workup [2,30] along with detailed anamnesis, diagnostic elimination diets, skin prick tests, and sIgE. Lack of OFC in all patients is explained by patients’ refusal to partake in it. When cow milk is the only suspected allergen and the only food in the diet, the diagnosis is simpler than in cases where they are already ingesting a variety of foods and OFC could not be a standard procedure. An oral food challenge test was performed in children with more than one food in their diet to confirm a positive diagnosis directly before initiation of OIT. Patients with negative OFC were actually patients with mild CMA that followed a rapid reintroduction of baked milk with an accelerated induction of tolerance.

Oral immunotherapy is a therapeutic method that permits the induction of tolerance and a normal diet after completion of it. OIT to milk is similar to peanut OIT regarding effectiveness in inducing clinical desensitization to the culprit allergen, but with a lower risk of allergic reactions during OIT. Clinical trial data are more limited, and there are no approved formulations for OIT. A significant challenge in determining the efficacy of several therapies for milk and egg allergies is that the natural rate of resolution of these allergies is much higher than for peanuts. In a 2012 meta-analysis of five trials that analyzed milk OIT (including 218 children), milk OIT increased the likelihood of developing full tolerance to milk by 10-fold compared to children without interventions. [17].

The quality of the allergen is critical for both OFC and OIT and may vary in commercial products; thus, it is hard to standardize this method [31]. In the present study, 88.6% of patients followed this procedure with a good response (only 11.1% of them had persistent CMA after 2-year follow up). More patients with an eviction diet who did not follow OIT presented persistent CMA at the end of the follow up period. Garcia-Ara et al. [32] also reported a high successful rate of desensitization after 1-year follow up (88–100%), depending on basal sIgE to milk. In the present study, the patients were not stratified according to basal evaluation of SPT and sIgE. A lower rate response was also mentioned by Kuitunen et al. (72%) after 6 months of OIT [33]. In another study from Denmark and Australia, patients achieved tolerance in a variable rate (28–77%) at the age of 2 for cow milk, without any interventions [34,35]. This difference could be explained by different inclusion criteria, duration of OIT, or a non-interventional attitude. The present analysis included all the patients with a positive diagnosis of CMA who presented for allergological evaluation. Increasing the duration of OIT and follow up may increase the success rate of it. We did not find associations between tolerance to milk and gender or other food allergies, in accordance with other published results [36,37]. This study sustained the role of OIT in obtaining tolerance, which may permit a normal diet independent from personal or familial history of allergy or from patient gender.

The reported rate of success and the less adverse events of OIT could be explained through a modified protocol of OIT, which used baked milk instead of raw milk until tolerance was induced. The children that obtained tolerance to baked milk after 6–9 months of OIT also tolerated dairy products as a component of the normal diet, or raw milk without any reactions after they switched from heated to unheated milk. Similar results were also reported by Esmaeilzadeh H et al. [38], who demonstrated that introducing baked milk products into the diet of patients with a milk allergy can accelerate the tolerance of unheated milk, but basal sIgE could not predict the success of OIT. Many concerns are raised regarding milk OIT because, unlike most other allergenic foods, milk is typically consumed in diverse forms several times per day, and a total daily dose that could be high may be not tolerated, especially in the presence of anaphylaxis co-factors [39]. Cow milk tolerance can spontaneously occur in the first years of life; thus, the faster tolerance we observed in most of the patients could be a consequence of both immune modulations via OIT with baked milk or may be due to a milder phenotype of CMA. However, this strategy induced a good rate of response to OIT in patients with anaphylaxis as a primary manifestation in the present study; thus, we may suppose that baked milk may accelerate, in a safe manner, the induction of tolerance to milk.

A double-blind placebo0controlled food challenge (DBPCFC) remains the gold standard for a positive diagnosis of CMA, but it is time consuming, expensive, can only be performed under medical guidance, only in specialized clinics, and it has a high risk of inducing severe anaphylactic reactions [9,30]. In addition, the quality of life of patients is affected when they experience a positive challenge test, and for this reason, may refuse to follow an OFC or oral immunotherapy [40]. Development of molecular biology in the last 10 years has permitted an increase in the accuracy of the diagnosis without referring the patient to a DBPCFC. Measurement of sIgE to different allergenic proteins from milk permits an identification of patterns of sensitization in complex polysensitized patients and is useful in identifying different phenotypes of CMA [41].

The present study showed that high levels of sIgE to milk and casein may predict the persistence of CMA despite oral immunotherapy. Previous data showed that patients with persistent CMA have higher values of sIgE to milk than those that can respond to oral immunotherapy [33,42,43], and it may predict the long-term outcome of milk OIT [20,42]. Kuitunen et al. also [33] demonstrated that high basal levels of sIgE to casein, alpha-lactalbumin and betalactoglobulin before the start of OIT were associated with a lower maintenance dose reached at the end of OIT. In addition, Savilahti EM et al. [44] reported that a high level of sIgE to milk and casein could predict a failure to achieve desensitization in milk OIT. It is also important to identify a value of sIge that might predict the resolution of CMA. We calculated a cut off value of 2.5 kU/L for sIgE to milk with 45.76% sensitivity and 100% specificity, but the size of the wheal in the skin prick test was not a predictive marker for OIT outcome. Yavuz ST et al. [45] reported that children with sIgE to milk below 6 kU/L outgrew CMA earlier than those with higher levels. In our cohort, the cut off value for sIgE was lower, but the outcome was to predict the resolution and not the interval of time after which we obtained it. 

Component-resolved diagnostics before OIT can help to identify children with a lower probability of a successful OIT outcome. sIgE to casein over 11.73 kU/L predicted a failure of achieving tolerance after OIT, with a sensitivity of 93% and a specificity of 60% in the present study. Kuitunen et al. [33] also reported that sIgE to milk allergens might have a better role in predicting resolution of CMA after OIT compared to other markers. It is essential to establish the role of these markers in order to identify candidates for OIT with a good resolution rate. Patient data derived from modern technology, in combination with a classical approach through the patient’s history, can be translated into patient-tailored interventions.

The main strength of the paper is that it presents a clinical and biological analysis of a cohort of patients with IgE-mediated CMA in Romania. The present study offers information from basal evaluation of patients with CMA that might predict the success of a medical intervention in those patients, allowing them to have a better quality of life. The study identifies some aspects that could be improved in the management of CMA. There are also some limitations of the study. First, OFC was not performed in all the patients to measure the exact amount of milk that produces clinical reactivity, and because of this reason, the OIT started in the same way in all included patients. Second, the evaluation of the children was performed at different ages not immediately after the onset of CMA. Most of the parents postponed the evaluation of their children until the moment of entrance in kindergarten or in school to see if they had a risk for severe reactions if an accidental exposure to milk might occur. Third, there was no control group in the present study. It would be interesting to have the possibility to evaluate the patients with mild forms of CMA and to compare the natural resolution of CMA with the active intervention (oral immunotherapy or rapid reintroduction of baked milk). Patients with mild forms of CMA were under pediatrician surveillance, and they followed an eviction diet, which is sometimes a long-term attitude, and they do not benefit from an active intervention. 

## 5. Conclusions

Anaphylaxis with only skin and mucosal involvement represents one of the most frequent manifestations in children with IgE-mediated CMA, although severe anaphylaxis may be present as an initial manifestation of CMA. Basal values of sIgE for milk and casein predict the occurrence of tolerance to milk after 2-year follow up in patients with CMA, including those with anaphylaxis as the first manifestation. OIT, or a rapid reintroduction with baked milk, may be used as an approach for CMA with IgE-mediated mechanisms, and it may result in the induction of tolerance faster and in a higher percentage of patients, allowing for a normal diet without any restrictions.

## Figures and Tables

**Table 1:** Protocol of rapid reintroduction of milk and oral immunotherapy to milk.

Phases	Rapid Reintroduction of Baked Milk		Oral Immunotherapy	
	Interval of Time between Dose Escalation	Amount of Baked Milk	Interval of Time between Dose Escalation	Amount of Baked Milk
Build up phase	30 min	1 mL	30 min	0.05 mL
	30 min	2 mL	30 min	0.1 mL
	30 min	4 mL	30 min	0.2 mL
	30 min	8 mL	30 min	0.4 mL
	30 min	16 mL	30 min	1 mL
	24 h	25 mL	30 min	2 mL
	48 h	50 mL	24 h	4 mL
			48 h	8 mL
			36 h	16 mL
			1 week	25 mL
			2 weeks	50 mL
Maintenance dose	1 week	100 mL	1 month	100 mL
	1 week	200 mL	3 months	200 mL

**Table 2:** Demographic data of patients with cow milk allergy.

Parameter		CMA (*n* = 70)	Patients without Tolerance (*n* = 10)	Patients with Tolerance (*n* = 60)	*p*
Gender	M	41 (58.6%)	8 (80%)	33 (55%)	0.178
	F	29 (49.4%)	2 (20%)	27 (45%)	
Family history of atopy		29 (41.4%)	2 (6.9%)	27 (83.1%)	0.166
Personal history of respiratory allergy		21 (30%)	3 (30%)	18 (30%)	0.918
Personal history of food allergy		20 (28.6%)	3 (30%)	17 (28.6%)	1
Living area	Urban	62 (88.6%)	10 (100%)	52 (83.9%)	0.591
	Non-urban	8 (11.4%)	0	8 (13.3%)	

**Table 3:** Primary clinical manifestation of CMA.

Parameter		Patients without Tolerance (*n* = 10)	Patients with Tolerance (*n* = 60)	*p*
Age of symptoms onset (months) *		9 (6–18)	9 (6–11.5)	0.060
Manifestations	Anaphylaxis	3 (14.3%)	18 (85.7%)	0.916
	Acute urticaria	2 (12.5%)	14 (87.5%)	
	Atopic dermatitis	3 (13%)	20 (87%)	
	Digestive symptoms	1 (33.3%)	2 (66.7%)	
	Urticaria + atopic dermatitis	1 (14.3%)	6 (85.7%)	

* Data are expressed as median and percentile.

**Table 4:** Basal results of skin test and laboratory values in patients with cow milk allergy.

Parameter.	Patients without Tolerance (*n* = 10)	Patients with Tolerance (*n* = 60)	*p*
Size of wheal (SPT)	8 (4.75–15.75)	5 (4–8)	0.08
sIgE to milk	12.77 (4.10–86.88)	3.2 (0.6–13.5)	0.039
sIgE to casein	14.3 (1.5–45.72)	0.96 (0.35–5.45)	0.01
sIgE to alpha-lactalbumin	2.3 (0.35–28.02)	2.1 (0.48–7.88)	0.926
sIgE to beta-lactoglobulin	2.42 (0.35–14.9)	1.5 (0.35–5.14)	0.755

Data are expressed as median and 25–27 percentiles.

**Table 5:** Results of oral food challenge test and correlation with acquired tolerance.

Parameter		Patients without Tolerance (*n* = 10)	Patients with Tolerance (*n* = 60)	*p*
OFC	Negative	1 (10%)	43 (71.67%)	0.003
	Positive	2 (20%)	16 (26.66%)	
	Not done	7 (70%)	1 (1.66%)	

**Table 6:** Basal results of skin test and laboratory values in patients with anaphylaxis induced by cow milk.

Parameter	Patients without Tolerance (*n* = 3)	Patients with Tolerance (*n* = 18)	*p*
Size of wheal (SPT)	15 (5)	7 (5–9)	0.185
sIgE to milk	91.66 (20)	5.3 (1.12–13.1)	0.019
sIgE to casein	74.3 (22.4)	2.3 (0.45–8.87)	0.017
sIgE to alpha-lactalbumin	78.2 (0.9)	2.1 (0.8–8.12)	0.221
sIgE to beta-lactoglobulin	21.8 (0.2)	0.58 (0.35–6.7)	0.534

Data are expressed as median and 25–27 percentiles.

**Table 7:** ROC curve analysis for oral tolerance at 2-year follow up.

Parameter	AUC	Cut-Off Value	Sensitivity	Specificity	*p*
sIgE to milk	0.705 (95% CI 0.550−0.860)	2.5 kU/l	45.7% (95% CI 32.7−59.2)	100% (95% CI 69.2−100.0)	0.012
sIgE to casein	0.755 (95% CI 0.581−0.93)	11.73 kU/l	93% (95% CI 83.8−98.2)	60% (95% CI 26.2−87.8)	0.005

## Data Availability

The data presented in this study are available on request from the corresponding author. The data are not publicly available due to privacy and ethical restrictions.
